# The SNP rs3128965 of HLA-DPB1 as a Genetic Marker of the AERD Phenotype

**DOI:** 10.1371/journal.pone.0111220

**Published:** 2014-12-23

**Authors:** Seung-Hyun Kim, Bo-Young Cho, Hyunna Choi, Eun-Soon Shin, Young-Min Ye, Jong-Eun Lee, Hae-Sim Park

**Affiliations:** 1 Department of Allergy & Clinical Immunology, Ajou University School of Medicine, Suwon, Korea,; 2 DNA link, Inc., Seoul, Korea; University of Birmingham, United Kingdom

## Abstract

**Background:**

Two common clinical syndromes of acetylsalicylic acid (aspirin) hypersensitivity, aspirin-exacerbated respiratory disease (AERD) and aspirin-exacerbated cutaneous disease (AECD), were subjected to a genome-wide association study to identify strong genetic markers for aspirin hypersensitivity in a Korean population.

**Methods:**

A comparison of SNP genotype frequencies on an Affymetrix Genome-Wide Human SNP array of 179 AERD patients and 1989 healthy normal control subjects (NC) revealed SNPs on chromosome 6 that were associated with AERD, but not AECD. To validate the association, we enrolled a second cohort comprising AERD (n = 264), NC (n = 238) and disease-control (aspirin tolerant asthma; ATA, n = 387) groups.

**Results:**

The minor genotype frequency (AG or AA) of a particular SNP, rs3128965, in the HLA-DPB1 region was higher in the AERD group compared to the ATA or NC group (*P* = 0.001, *P* = 0.002, in a co-dominant analysis model, respectively). Comparison of rs3128965 alleles with the clinical features of asthmatics revealed that patients harboring the A allele had increased bronchial hyperresponsiveness to inhaled aspirin and methacholine, and higher 15-HETE levels, than those without the A allele (*P* = 0.039, 0.037, and 0.004, respectively).

**Conclusions:**

This implies the potential of rs3128965 as a genetic marker for diagnosis and prediction of the AERD phenotype.

## Introduction

Hypersensitivity reactions to aspirin manifest as broad-ranging clinical features of allergic reactions including aspirin-exacerbated respiratory disease (AERD), aspirin-exacerbated cutaneous disease (AECD), and occasionally, anaphylaxis [1], [2]. A genetic association study reported several genetic risk factors for aspirin hypersensitivity, with most being involved in arachidonate metabolism or the inflammatory pathway [3], [4] To date, the best genetic marker for AERD is HLA-DPB1*0301, which is also associated with a higher prevalence of chronic rhinosinusitis and a higher leukotriene receptor antagonist dose to control asthmatic symptoms in AERD patients [5]–[7].

To identify additional genetic determinants for increased risk of developing aspirin hypersensitivity in adult patients with underlying asthma or urticaria, we performed a genome-wide association study (GWAS) using an Affymetrix Genome-Wide Human SNP array.

To verify any significant associations, a second replication study was carried out using a larger study cohort in a Korean population.

Here we report a newly identified single-nucleotide polymorphism (SNP), rs3128965, as a genetic marker of the AERD phenotype.

## Methods

### Study subjects

Patients with aspirin hypersensitivity (n = 390; 179 AERD, 211 AECD) were recruited from the Ajou University Medical Center, Suwon, Korea. AERD patients were diagnosed by a positive lysine-aspirin bronchoprovocation test (L-ASA BPT) from asthmatic patients who showed typical clinical features of asthma [8]. Lys-ASA BPT was performed as described previously with increasing dosages of Lys-ASA (75 mg, 150 mg, 300 mg) [8]. The AERD patients had upper and lower respiratory symptoms without any history of drug-induced cutaneous symptoms, while the ATA patients were defined as asthmatic patients showing a negative response to L-ASA BPT. The AECD patients were defined as having > 6 weeks of daily itchy wheals, a history of chronic urticaria/angioedema aggravated by ingestion of more than two nonsteroidal anti-inflammatory drugs, and showing positive responses in the oral aspirin challenge test. Blood samples and clinical information from patients and controls were collected with written informed consent and with the approval of the Ethics Review Board of the Ajou Medical Center (AJIRB-GEN-GEN-11-304).

Clinical characterization of AERD and AECD patients including rhinosinusitis, pulmonary functional test, and aspirin provocation test were determined as described previously [9]. Serum total IgE and eosinophil cationic protein (ECP) levels were measured using the ImmunoCAP system (Phadia, AB, Uppsala, Sweden). The presence of rhinosinusitis was determined based on paranasal sinus X-rays, CT scans, and/or rhinoscopy. The presence of moderate to chronic rhinosinusitis was defined as a Lund-Mackay score higher than 3 [10]. The 15-hydroxyeicosatetraenoic acid (15-HETE) level was measured in the cell supernatants of peripheral blood leukocytes treated with arachidonic acid (40 µM) at 37°C for 5 min, as described previously [11], [12], and quantified using an EIA kit (Cayman Chemicals, Ann Arbor, MI, USA).

### Genome-wide association and the replication studies

Using an Affymetrix genome-wide human SNP array, a GWAS was performed to identify the genetic risk factors for aspirin hypersensitivity in adult patients with underlying asthma or urticaria (AERD, n = 179; AECD, n = 211). Anonymous genotype data from 1989 healthy normal control subjects (NC) were supplied by the Korean National Institute of Health (http://www.cdc.go.kr). The clinical characteristics of the study subjects used in the genome-wide association study are listed in S1 Table.

For the initial analysis of the unrelated case-control samples, we examined the identity-by-state (IBS) counts of the whole-genome genotype data, filtered out samples with <97% genotype call rate, and excluded samples that shared >10% of their genome. There was no evidence of differential genotyping between patients and controls, and no evidence of a population substructure in either group (data not shown). We used the Bayesian Robust Linear Modeling with Mahalanobis distance classifier (BRLMM) genotype calling algorithm to assess the SNPs. SNP markers with low genotyping call rates (<97%), low minor-allele frequencies (<1%) and deviation from Hardy-Weinberg equilibrium (*P*<0.0001) were filtered out. The association between each SNP and the case-control was assessed using a Cochran–Armitage trend test using PLINK (v1.07) [13]. The quantile-quantile (Q–Q) plot was plotted with the expected distribution of association test statistics under null distribution across the Cochran– Armitage trend observed *P*-values with *R* statistical environment version 2.9.0. A Manhattan plot was generated using PLINK. Q-Q plots are shown in S1 Fig. (AERD vs. NC) and S2 Fig. (AECD vs. NC).

Targeted SNP genotyping was also performed using a larger study cohort composing AERD (n = 264), ATA (n = 387), and NC groups (n = 238). The clinical characteristics of the study subjects used in the second-stage validation study are listed in S2 Table. SNP genotyping was performed by the primer extension method using a SNAPshot ddNTP primer extension kit (Applied Biosystems, Foster City, CA, USA). The sequences of the amplifying and extension primers were as follows: forward, 5′-AGCCTCATTTCCTCAAAAA-3′; reverse, 5′-ACAAGCAGGAAAGAGAATGA-3′; and extension, 5′-AGGAAACTTGTGAGAAACCTATGCA-3′. The genotype distributions of the AERD patients and the control subjects in the second-stage replication analyses were assessed using multiple logistic regression models (dominant, recessive and co-dominant), with age and gender as covariates. All statistical analyses were performed using SPSS, version 12.0 (SPSS, Chicago, IL, USA).

## Results

After filtering for study subjects with an average call rate of 97%, and markers with high genotyping call rates (>97%), high minor allele frequencies (>1%) and no deviation from Hardy-Weinberg equilibrium (*P*>0.0001), we included genotype data from 275,862 SNPs from 2379 study subjects (179 AERD, 211 AECD, 1989 NC). The mean age of AERD patients was 43.72 years (±13.09), that of AECD patients was 36.09 years (±11.14), and that of healthy normal controls was 47.67 years (±7.13) (S1 Table). There was a significant difference between the mean age of the case and NC groups (<0.001), but no significant difference in gender proportions between the two groups.

**Figure 1:**
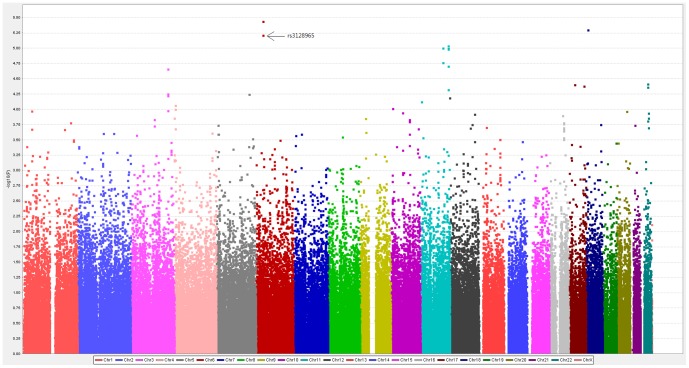
A genome-wide Manhattan plot of GWAS in a comparison of AERD with NC. Distribution of negative logarithm *P* values are plotted against chromosomes.

A genome-wide Manhattan plot was drawn using the chromosomal positions of individual SNPs (*x*-axis) and the negative logarithm of *P* values calculated using the Cochran–Armitage trend test (*y*-axis; Fig. 1). The top 20 SNPs associated with AERD are shown in Table 1. A genome-wide Manhattan plot of GWAS in a comparison of AECD with NC is shown in S3 Fig. The top 20 SNPs associated with AERD are shown in S3 Table.

**Table 1:** Genome-wide association result for the top 20 single-nucleotide polymorphisms (SNPs) in a comparison of AERD with NC.

SNP	Chr	Physical Location	Gene	Location	MAF		*P* value§
					AERD (n = 179)	NC (n = 1989)	
rs3117230	6p21.32	33183613		intergenic	0.16	0.08	3.61E–06
rs751355	18p11.22	9418420		intergenic	0.46	0.34	4.98E–06
rs3128965	6p21.32	33163877	HLA-DPB1	downstream	0.16	0.08	6.06E–06
rs12576973	11q24.2	126763852		intergenic	0.22	0.14	9.10E–06
rs10895516	11q22.3	103113180		intergenic	0.30	0.20	9.89E–06
rs12577873	11q24.2	126767198		intergenic	0.22	0.14	1.00E–05
rs2618221	11q24.2	126757104		intergenic	0.22	0.14	1.03E–05
rs17101477	11q22.3	103111948		intergenic	0.30	0.20	1.70E–05
rs2697717	11q24.2	126709174		intergenic	0.25	0.16	1.96E–05
rs1343036	3q26.2	170724940	MECOM	Intron	0.23	0.15	2.17E–05
rs5755393	22q12.3	33607037	ISX	intergenic	0.38	0.50	3.80E–05
rs16968024	17q11.2	28465300	ACCN1	Intron	0.15	0.08	3.90E–05
rs477963	17q25.1	70153035		intergenic	0.16	0.10	4.13E–05
rs2038088	22q12.3	33595255		intergenic	0.38	0.50	4.32E–05
rs10893679	11q24.2	126786589		intergenic	0.22	0.14	4.75E–05
rs12495069	3q26.2	170703798	MECOM	Intron	0.24	0.16	5.58E–05
rs6579884	5q33.1	151016491		intergenic	0.14	0.08	5.63E–05
rs6444857	3q26.2	170721406	MECOM	Intron	0.23	0.15	5.90E–05
rs904664	12p13.33	99608	IQSEC3	5'UTR	0.06	0.03	6.46E–05
rs7934354	11p15.4	5522482	OR52H1	Exon	0.29	0.40	7.44E–05

§ Cochran–Armitage trend test.

Abbreviations: AERD, aspirin-exacerbated respiratory disease; NC, normal controls, MAF, minor allele frequency; HLA-DPB1, major histocompatibility complex, class ii, dp beta 1; ACCN1, amiloride-sensitive cation channel 1; MECOM; MDS1 and EVI1 complex locus; IQSEC3, iq motif and sec7 domain 3, OR52H1, olfactory receptor, family 52, subfamily h, member 1.

**Figure 2:**
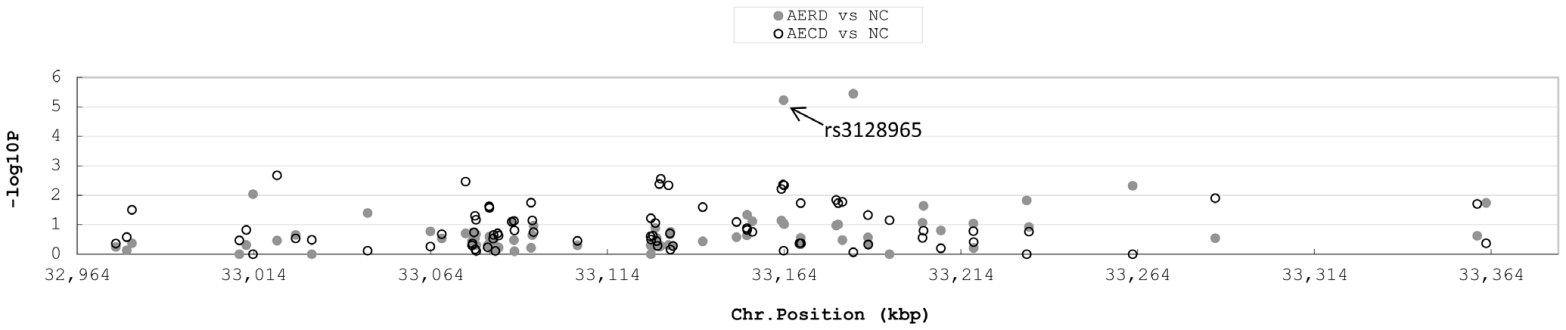
SNP rs3128965 of HLA-DPB1 as a target SNP for susceptibility to AERD. Negative logarithm *P* values are shown around the HLA-DPB1 region. Closed circles indicate the *P* value of each SNP in a comparison of AERD and NC. Open circles indicate the *P* value of each SNP in a comparison of AECD and NC.

Two SNPs located on chromosome 6, rs3117230 and rs3128965, showed a strong association with the AERD phenotype and were found to be in perfect linkage disequilibrium (D′ = 0.984, r^2^ = 0.961). However, none of SNPs listed in Table 1 reached significance at a genome-wide threshold after Bonferroni multiple correction (*P*<1.8×10^−7^). The SNP rs3128965, located in the HLA-DPB1 region of chromosome 6, was targeted for a second-stage replication study with a larger cohort due to its specific association with the AERD phenotype, but not the AECD phenotype (Fig. 2). The genome-wide significance of rs3128965 was replicated in the second study cohort (Table 2). The genotype frequency of the minor allele (AG or AA) was significantly higher in the AERD group compared to the disease control (ATA) and NC groups (*P* = 0.001, *P* = 0.002, in a co-dominant analysis model, respectively). The significance remained after multiple correction testing.

**Table 2:** Genetic association of the SNP, rs3128965, with AERD phenotype.

	Discovery GWAS				Replication study								
	AIA	NC	AERD vs. NC		AERD	ATA	NC	AERD vs. ATA		AERD vs. NC		ATA vs. NC	
	(n = 179)	(n = 1989)	*P* Value§	OR(95% CI)¶	(n = 264)	(n = 387)	(n = 238)	*P* value§	OR(95% CI)¶	*P* value§	OR(95% CI)¶	*P* value§	OR(95% CI)¶
**GG**	129(72.1%)	1664(83.7%)	**1.40E–05**	1(reference)	191(72.35%)	323(83.46%)	195(81.93%)	**0.001**	1(reference)	**0.002**	1(reference)	0.646	1(reference)
**AG**	44(24.6%)	313(15.7%)	**2.16E–03**	1.860(1.287, 2.689)	65(24.62%)	60(15.50%)	43(18.07%)	0.098	1.805(1.216, 2.680)	0.999	1.631(1.021, 2.604)	0.999	0.769(0.499, 1.268)
**AA**	6(3.3%)	12(0.6%)	**9.70E–05**	5.747(2.027, 16.293)	8(3.03%)	4(1.03%)	0(0%)	**0.001**	3.166(0.937, 10.703)	**0.008**	NA	0.462	NA

Abbreviations: AERD, aspirin-exacerbated respiratory disease; ATA, aspirin-tolerant asthma; NC, normal controls; NA; not applicable.

§ Each *P*-value was calculated for a co-dominant, dominant and recessive model. Logistic regression analysis was applied to control for age and gender as covariates.

¶ Odd ratio was given by co-dominant analysis model. Bold character indicates significance.

The clinical features associated with this SNP are shown in Table 3. Adult asthmatic patients with risk allele A showed enhanced bronchial hyperresponsiveness to lysine-aspirin inhaled (% fall of FEV after L-ASA BPT) and methacholine (PC_20_), and higher 15-HETE levels than those without the A allele (*P* = 0.039, *P* = 0.037 and *P* = 0.004, respectively; Table 3).

**Table 3:** Clinical features according to the SNP, rs3128965, in asthmatics (264 AERD and 387 ATA).

	rs3128965		
	GG (n = 514)	AA+AG (n = 137)	*P* value§
**Age (year)**	43.77±14.31	43.74±13.56	0.985
**Sex (female, %)** ¶	303/514 (58.9%)	95/137 (69.3%)	**0.030**
**Atopy (presence, %)** ¶	234/425 (55.1%)	70/114 (61.4%)	0.243
**Total IgE (IU/mL)**	390.59±741.4	336.89±413.41	0.480
**PC_20_, methacholine (mg/mL)**	9.64±19.2	6.09±10.24	**0.037**
**Base line FEV1 (%)**	87.09±18.65	88.02±18.69	0.806
**% Fall of FEV1 by aspirin challenge**	15.92±11.04	20.45±14.77	**0.039**
**Chronic rhinosinusitits (LM score 3-4, %)** ¶	52/96 (54.2%)	24/33 (72.7%)	0.068
**Total eosinophil count (count/µl)**	455.34±940.01	486.18±638.24	0.763
**ECP (µg/L)**	30.8±36.98	34.38±36.12	0.470
**Sputum_eosinophil (%)**	22.19±34.14	26.66±37.55	0.430
**Sputum_neutrophil (%)**	57.02±33.65	59.83±34.33	0.683
**15-HETE (pg/mL)** *	3031.9±965.3	3815.2±1316.1	**0.004**

Abbreviations: FEV1, forced expiratory volume in 1 s; IgE, immunoglobulin E; methacholine PC_20_, the provocative concentration of methacholine required to cause a 20% fall in FEV1; 15-HETE, 15-hydroxyeicosatetraenoic acid; ECP, eosinophil cationic protein; LM score, Lund-Mackay CT score. Values are given as n (%) for categorical variables and as mean ± SD for continuous variables.

¶ count number/valid number.

*median ± std.

§ *P* value were applied by Fisher exact test for categorical variable and T-test for continuous variable.

Bold character indicates significance.

## Discussion

Several genetic studies on AERD have been conducted [3], [4] and the HLA-DPB1*0301 was reported to have a strong association with the AERD phenotype [5]. The association of DPB1*0301 with AERD was also identified in a Korean population, together with a significant association with the requirement for a leukotriene receptor antagonist [6], [7]. The HLA haplotype HLA-DRB1*1301–DQB1*0609 was identified as a genetic risk factor for AECD in Koreans [14]. Various immunological and genetic mechanisms may contribute to the development of the clinical features of aspirin hypersensitivity.

In this study, rs3128965 was identified as a genetic marker for AERD in an initial GWAS, and its association was validated significance in a subsequent replication study. Recently, two GWAS suggested two novel SNPs, rs7572857 of CEP68 [15] and rs1042151 of HLA-DPB1 [16] as genetic markers for AERD susceptibility in a Korean population. *CEP68* rs7572857 was not associated with the AERD phenotype in the present study cohort, while HLA-DPB1 rs1042151 was in perfect linkage disequilibrium with rs3128965, the target SNP in the present study. The replicated association of HLA-DPB1 gene polymorphisms with AERD phenotype suggests that the HLA-DPB1 gene has an important role in AERD susceptibility. Moreover, this study identified the specific influence of HLA-DPB1 rs3128965 on the susceptibility to AERD, but not AECD, suggesting a different genetic mechanism involved in the pathogenesis of AECD.

In addition, rs3128965 showed a significant association with the percentage decrease in FEV1 after L-ASA BPT, which is the gold standard for diagnosis of AERD [17]. This implies that rs3128965 is a reliable genetic marker for diagnosis of aspirin hypersensitivity in adult asthmatic patients.

The significant association of rs3128965 with increased 15-HETE levels, a potential biomarker for AERD patients for *in vitro* diagnosis [12], [18], and enhanced airway hyperresponsiveness, methacholine PC_20_, demonstrated that this SNP could be useful in predicting the progression of asthmatic symptoms in AERD patients after exposure to aspirin.

AERD patients are more likely to present with severe or difficult-to-treat asthma because of poor lung function, life threatening exacerbation, and higher requirements for therapeutics such as steroid and leukotriene receptor antagonists (LTRAs) [19], [20]. A previous report suggested use of the HLA-DPB1*0301 marker for predicting LTRA requirement in AERD patients [7]. In the present study, rs3128965 was associated with LTRA requirement (S4 Table), suggesting that this SNP could be a potential marker for predicting favorable response to LTRA treatment in AERD patients.

This is the first GWAS to demonstrate differential genetic susceptibility to two different clinical phenotypes, AERD and AECD. However, this study does have a number of limitations. First, the sample size was relatively small for a GWAS. To exclude false positive associations, follow-up targeted SNP genotyping was performed. However, other candidate SNPs detected on the initial GWAS also require validation by replication studies. Second, this study did not identify any novel genes suggested to confer genetic susceptibility to aspirin, and the limited sample size could not guarantee target SNPs with genome-wide significance. Therefore future replication studies are required.

Taken together, our results validate the use of rs3128965 as a genetic marker to predict the AERD phenotype, both *in vivo* (greater bronchoconstriction to aspirin exposure) and *in vitro* (greater production of 15-HETE from peripheral blood cells). These findings also confirm the usefulness of SNP genotyping for diagnosis and prediction of the AERD phenotype.

## Supporting Information

S1 Fig **Q-Q plot of GWAS data from the patients with AERD** ***vs.*** **NC.**
(TIF)

S2 Fig **Q-Q plot of GWAS data from the patients with AECD** ***vs*** **. NC.**
(TIF)

S3 Fig **The Manhattan plot of GWAS shows** ***P*** **values for AECD** ***vs.*** **NC using a Cochran–Armitage trend test.**
(TIF)

S1 Table **Clinical demographics of the study subjects enrolled in the first genome-wide association study.**
(DOCX)

S2 Table **Clinical demographics of the study subjects enrolled in the second validation study.**
(DOCX)

S3 Table **Genome-wide association result for the top 20 single-nucleotide polymorphisms (SNPs) in a comparison of AECD and NC.**
(DOCX)

S4 Table **Association of the SNP rs3128965 with leukotriene receptor antagonist (LTRA) requirement in 75 AERD patients.**
(DOCX)
