# Supplementary figures and images for: The SNP rs3128965 of HLA-DPB1 as a Genetic Marker of the AERD Phenotype
Source: PLoS One. 2014 Dec 23;9(12):e111220. doi: 10.1371/journal.pone.0111220 (PMC4275175; doi:10.1371/journal.pone.0111220)

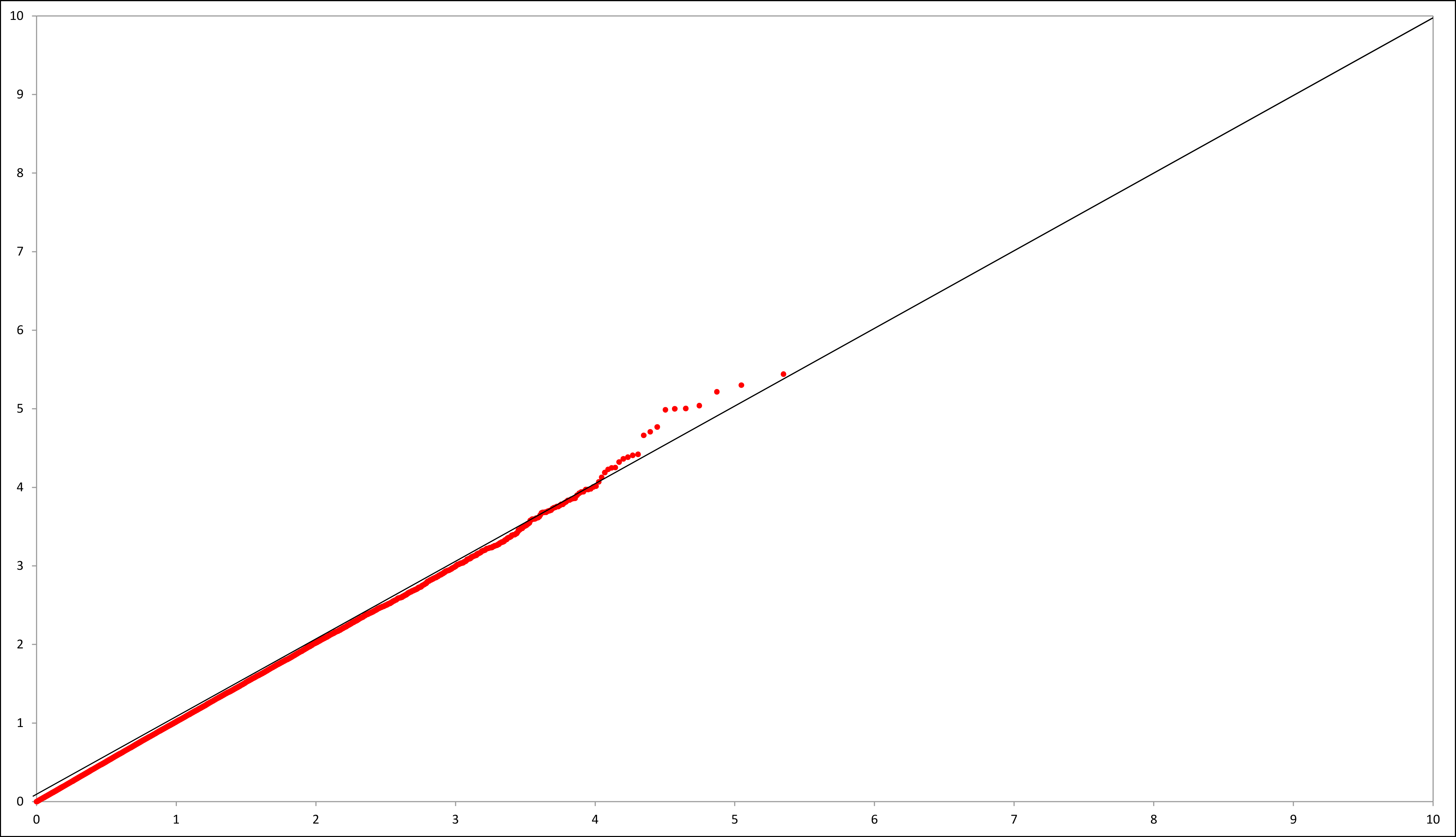

Supplement: S1 Fig — Q-Q plot of GWAS data from the patients with AERD vs. NC. (TIF) [file pone.0111220.s001.tif]

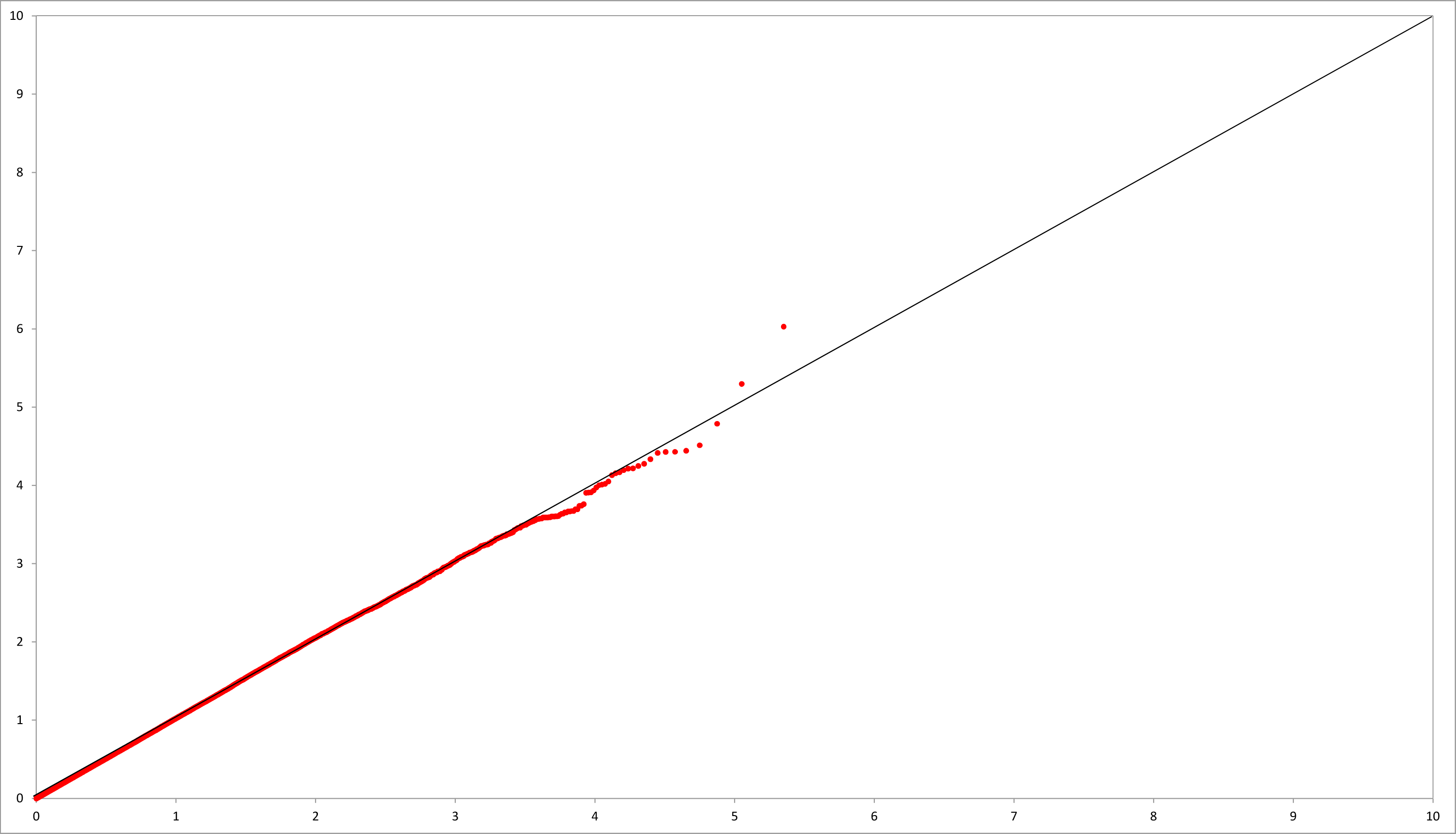

Supplement: S2 Fig — Q-Q plot of GWAS data from the patients with AECD vs . NC. (TIF) [file pone.0111220.s002.tif]

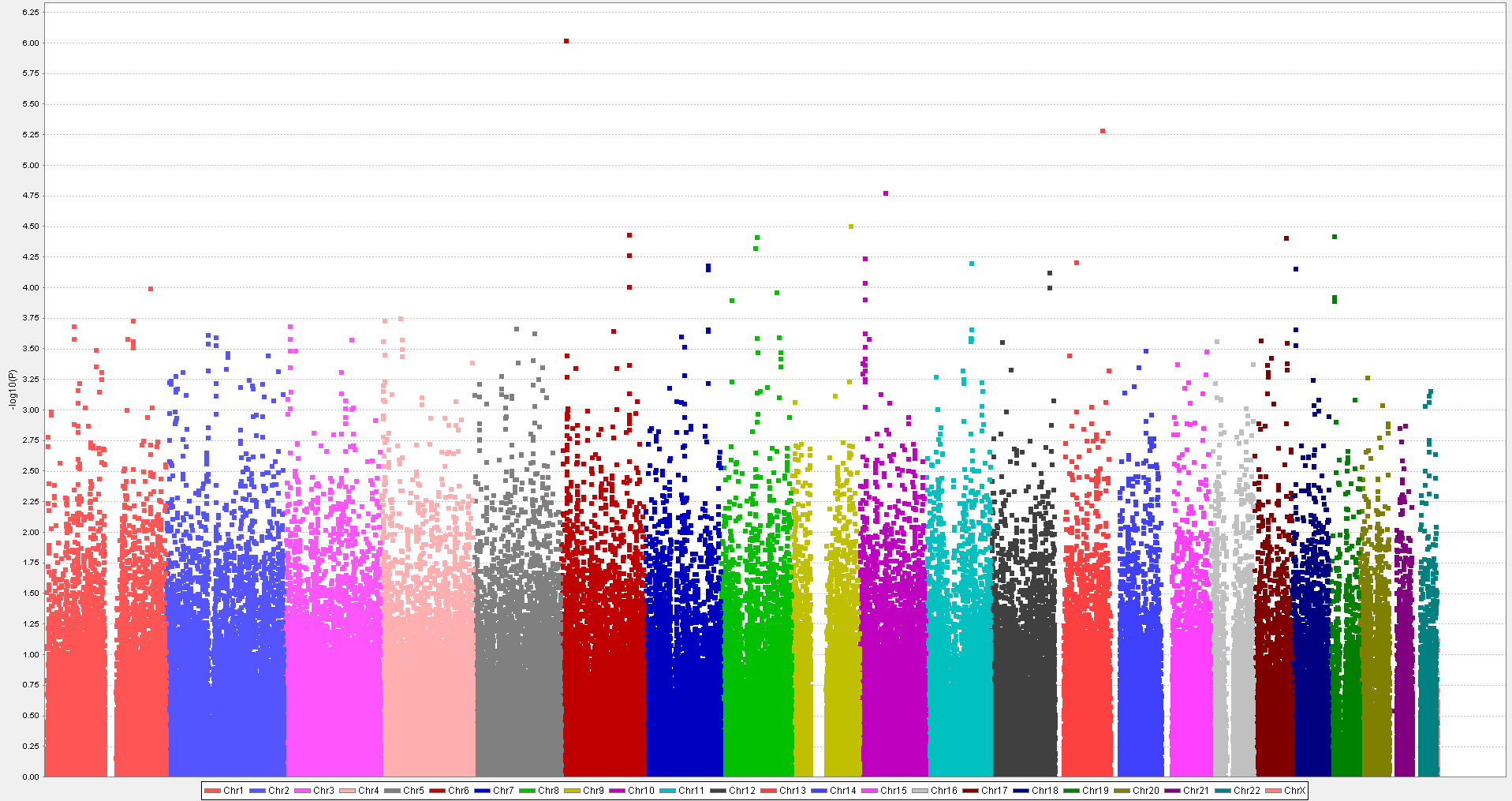

Supplement: S3 Fig — The Manhattan plot of GWAS shows P values for AECD vs. NC using a Cochran–Armitage trend test. (TIF) [file pone.0111220.s003.tif]
