# Supplementary material for: The SNP rs3128965 of HLA-DPB1 as a Genetic Marker of the AERD Phenotype
Source: PLoS One. 2014 Dec 23;9(12):e111220. doi: 10.1371/journal.pone.0111220 (PMC4275175; doi:10.1371/journal.pone.0111220)
Supplement: S1 Table — Clinical demographics of the study subjects enrolled in the first genome-wide association study. (DOCX) [file pone.0111220.s004.docx]

| **Table S1. Clinical demographics of the study subjects enrolled in the first genome-wide association study** | | | | | |
| --- | --- | --- | --- | --- | --- |
|  | **Control** |  | **Case** | | |
|  | **NC** |  | **AERD** |  | **AECD** |
|  | (n=1989) |  | (n=179) |  | (n=211) |
| **Age (year)** | 47.70±7.05/1989 |  | 43.72±13.09/179 |  | 36.09±11.14/211 |
| **Sex (female, %)**^¶^ | 1192/1989(59.9%) |  | 116/179(64.8%) |  | 122/211(57.8%) |
| **Atopy (presence, %)**^¶^ |  |  | 88/162(54.3%) |  | 118/198(59.6%) |
| **Total IgE (IU/mL)** |  |  | 324.38±398.77 |  | 268.95±290.12 |
| **Disease_period(year)*** |  |  | 6.38±5.95 |  | 3.66±5.77 |
| **Base line FEV1 (%)** |  |  | 84.99±16.29 |  |  |
| **PC_20_, methacholine (mg/mL)** | |  | 5.58±13.74 |  |  |
| **Chronic rhinosinusitits (LM score 3-4, %)^¶^** | |  | 36/48(75%) |  |  |

Abbreviations: FEV1, forced expiratory volume in 1 s; IgE, immunoglobulin E; methacholine PC_20_, the provocative concentration of methacholine required to cause a 20% fall in FEV1; AERD, aspirin-exacerbated respiratory disease; LM score, Lund-Mackay CT score ; AECD, aspirin-exacerbated cutaneous disease; NC, normal controls.

Values are given as n (%) for categorical variables and as mean ± SD for continuous variables. ^¶^ count number/valid number
