# Supplementary material for: The SNP rs3128965 of HLA-DPB1 as a Genetic Marker of the AERD Phenotype
Source: PLoS One. 2014 Dec 23;9(12):e111220. doi: 10.1371/journal.pone.0111220 (PMC4275175; doi:10.1371/journal.pone.0111220)
Supplement: S2 Table — Clinical demographics of the study subjects enrolled in the second validation study. (DOCX) [file pone.0111220.s005.docx]

**Table S2. Clinical demographics of the study subjects enrolled in the second validation study.**

|  | **AERD** | **ATA** | **NC** |  |
| --- | --- | --- | --- | --- |
|  | (n=264) | (n=387) | (n=238) |  |
| **Age (year)** | 43.36±13.56 | 44.04±14.54 | 33.01±13.12 |  |
| **Sex (female, %)** ^¶^ | 170/264(64.4%) | 228/387(58.9%) | 117/238(49.2%) |  |
| **Atopy (presence, %)**^¶^ | 129/242(53.3%) | 175/297(58.9%) | 21/200(10.5%) |  |
| **Total IgE (IU/mL)** | 395.67±628.86 | 368.02±729.73 | 93.74±170.85 |  |
| **PC_20_, methacholine (mg/mL)** | 6.98±17.56 | 10.74±17.87 | - |  |
| **Baseline_FEV1 (%)** | 86.74±18.24 | 90.36±20.67 | - |  |
| **% Fall of FEV1 by lysine aspirin challenge** | 22.93±11.12 | 7.01±4.61 | - |  |
| **Chronic rhinosinusitits (LM score 3-4, %)^¶^** | 61/92(66.3%) | 15/37(40.5%) |  |  |
| **Total eosinophil count ( count/μl)** | 432.89±415.82 | 480±1096.04 | - |  |
| **ECP (μg/L)** | 29.61±31.43 | 33.44±41.07 | - |  |
| **Sputum_eosinophil (%)** | 24.13±35.65 | 21.32±33.47 | - |  |
| **Sputum_neutrophil (%)** | 57.18±34.4 | 58.19±32.76 | - |  |
| **15-HETE (pg/mL)*** | 3366.61±1246.39 | 3031.62±833.27 | 2948.44±1214.25 |  |
| Abbreviations: AERD, aspirin-exacerbated respiratory disease; ATA, aspirin-tolerant asthma; NC, normal controls; FEV1, forced expiratory volume in 1 s; IgE, immunoglobulin E; methacholine PC_20_, the provocative concentration of methacholine required to cause a 20% fall in FEV1; 15-HETE, 15-hydroxyeicosatetraenoic acid; ECP, eosinophil cationic protein; LM score, Lund-Mackay CT score. Values are given as n (%) for categorical variables and as mean ± SD for continuous variables. ^¶^ count number/valid number *median±std | | | | |
