# Supplementary material for: The SNP rs3128965 of HLA-DPB1 as a Genetic Marker of the AERD Phenotype
Source: PLoS One. 2014 Dec 23;9(12):e111220. doi: 10.1371/journal.pone.0111220 (PMC4275175; doi:10.1371/journal.pone.0111220)
Supplement: S3 Table — Genome-wide association result for the top 20 single-nucleotide polymorphisms (SNPs) in a comparison of AECD and NC. (DOCX) [file pone.0111220.s006.docx]

| **Table S3. Genome-wide association result for the top 20 single-nucleotide polymorphisms (SNPs) in a comparison of AECD and NC** | | | | | | |  |
| --- | --- | --- | --- | --- | --- | --- | --- |
| **SNP** | **Chr** | **Physical Location** | **Gene** | **Location** | **MAF** | | ***P* value**^§^ |
|  |  |  |  |  | **AECD** (n=211) | **NC**  (n=1989) |  |
| rs1099 | 6p24.1 | 11210857 | HERV-FRD | 3'UTR | 0.24 | 0.36 | 9.34E-07 |
| rs12872448 | 13q32.3 | 98393248 | DOCK9 | Intron | 0.18 | 0.11 | 5.04E-06 |
| rs11000713 | 10q21.1 | 53507297 | PRKG1 | Intron | 0.14 | 0.08 | 1.63E-05 |
| rs10984711 | 9q33.1 | 121620396 |  | intergenic | 0.05 | 0.02 | 3.07E-05 |
| rs632430 | 6q24.1 | 140289814 |  | intergenic | 0.39 | 0.49 | 3.60E-05 |
| rs8101160 | 19p13.2 | 11634797 |  | intergenic | 0.20 | 0.30 | 3.71E-05 |
| rs10957648 | 8q21.11 | 74279370 |  | intergenic | 0.36 | 0.27 | 3.74E-05 |
| rs2241171 | 17q24.3 | 67582386 |  | intergenic | 0.10 | 0.19 | 3.84E-05 |
| rs16936692 | 8q13.3 | 71149834 |  | intergenic | 0.29 | 0.39 | 4.60E-05 |
| rs4896494 | 6q24.1 | 140238099 |  | intergenic | 0.34 | 0.45 | 5.30E-05 |
| rs7087075 | 10p14 | 10015749 |  | intergenic | 0.26 | 0.35 | 5.64E-05 |
| rs8000903 | 13q14.11 | 43858248 | SERP2 | Intron | 0.35 | 0.26 | 6.04E-05 |
| rs624184 | 11q21 | 93974725 | PIWIL4 | Exon | 0.14 | 0.08 | 6.10E-05 |
| rs10954390 | 7q32.3 | 132194395 | CHCHD3 | Intron | 0.16 | 0.10 | 6.37E-05 |
| rs9949325 | 18p11.22 | 8631331 | RAB12 | downstream | 0.28 | 0.38 | 6.81E-05 |
| rs7799524 | 7q32.3 | 132213794 | CHCHD3 | Intron | 0.16 | 0.10 | 6.96E-05 |
| rs7971538 | 12q24.31 | 120958046 | BCL7A | Intron | 0.11 | 0.06 | 7.38E-05 |
| rs17146745 | 10p14 | 10007525 |  | intergenic | 0.27 | 0.37 | 8.89E-05 |
| rs1028469 | 6q24.1 | 140305036 |  | intergenic | 0.32 | 0.42 | 9.52E-05 |
| rs11043285 | 12q24.31 | 120927917 | WDR66 | downstream | 0.11 | 0.06 | 9.71E-05 |
| ^§^ Cochran–Armitage trend test. | |  |  |  |  |  |  |
| Abbreviations: AERD, aspirin-exacerbated respiratory disease; NC, normal controls, MAF, minor allele frequency; HERV-FRD, HERV-FRD provirus ancestral Env polyprotein; DOCK9, dedicator of cytokinesis 9; PRKG1, protein kinase, cgmp-dependent, type I; SERP2, stress-associated endoplasmic reticulum protein family member 2; PIWIL4, piwi-like 4; CHCHD3, coiled-coil-helix-coiled-coil-helix domain containing 3; RAB12, coiled-coil-helix-coiled-coil-helix domain containing 3; BCL7A, b-cell cll/lymphoma 7a; WDR66, WD repeat domain 66. | | | | | | | |
