# Supplementary material for: The SNP rs3128965 of HLA-DPB1 as a Genetic Marker of the AERD Phenotype
Source: PLoS One. 2014 Dec 23;9(12):e111220. doi: 10.1371/journal.pone.0111220 (PMC4275175; doi:10.1371/journal.pone.0111220)
Supplement: S4 Table — Association of the SNP rs3128965 with leukotriene receptor antagonist (LTRA) requirement in 75 AERD patients. (DOCX) [file pone.0111220.s007.docx]

|  | | | | | | | |
| --- | --- | --- | --- | --- | --- | --- | --- |
| **Table S4. Association of the SNP rs3128965 with leukotriene receptor antagonist (LTRA) requirement in 75 AERD patients.** | | | | | |  |  |
| **Genotype** | **Group I*** | **Group II** | **Group III** | ***P* value**^§^ | | |  |
|  | (n= 20) | (n=33 ) | (n=22 ) | Group I vs. Group II | Group I vs. Group III | |  |
| **GG** | 9(45.0%) | 26(78.8%) | 19(86.4%) | 0.017 | 0.008 | |  |
| **AA+AG** | 11(55.0%) | 7(21.2%) | 3(13.6%) |  |  | |  |
| * Group I: LTRA requirement ≥200 mg/month , Group II: 5–150 mg/month, Group III: <5 mg/month | | | | |  | |  |

^§^Fisher exact test
